# Transforming health care systems towards high-performance organizations: qualitative study based on learning from COVID-19 pandemic in the Basque Country (Spain)

**DOI:** 10.1186/s12913-024-10810-w

**Published:** 2024-03-21

**Authors:** Ane Fullaondo, Irati Erreguerena, Esteban de Manuel Keenoy

**Affiliations:** Kronikgune Institute for Health Services Research, Barakaldo, Spain

**Keywords:** Healthcare system transformation, High-quality care, Multidimensional reform

## Abstract

**Background:**

The COVID-19 pandemic is one of the worst health catastrophes of the last century, which caused severe economic, political, and social consequences worldwide. Despite these devastating consequences, lessons learned provide a great opportunity that can drive the reform of health systems to become high-performing, effective, equitable, accessible, and sustainable organisations. This work identifies areas in which changes must be encouraged that will enable health systems to deal effectively with current and future challenges, beyond COVID-19.

**Methods:**

A realist design was chosen, based on qualitative data collection techniques, content analysis and triangulation to identify key domains of organizational interventions behind the changes implemented to react to the COVID-19 pandemic in the Basque Country. Twenty key informants were used as an expert source of information. Thematic analysis was done using the Framework Method.

**Results:**

The analysis of the interviews resulted in the identification of 116 codes, which were reviewed and agreed upon by the researchers. Following the process of methodological analysis, these codes were grouped into domains: seven themes and 23 sub-themes. Specifically, the themes are: responsiveness, telehealth, integration, knowledge management, professional roles, digitisation, and organisational communication. The detailed description of each theme and subtheme is presented.

**Conclusions:**

The findings of this work pretend to guide the transformation of health systems into organisations that can improve the health of their populations and provide high quality care. Such a multidimensional and comprehensive reform encompasses both strategic and operational actions in diverse areas and requires a broad and sustained political, technical, and financial commitment.

**Supplementary Information:**

The online version contains supplementary material available at 10.1186/s12913-024-10810-w.

## Background

The 2019 coronavirus disease pandemic (COVID-19) is one of the worst health catastrophes of the last century; it has caused devastating effects on the health of populations in some countries and severe economic, political, and social consequences in many more [1]. In all countries, regardless of income level, the pandemic has had a direct impact on health systems. Specifically in the Basque Country, 761,000 confirmed cases of COVID-19 and 7,049 related deaths are registered as of July 2022 [2]. 

Today, in the wake of the pandemic, two types of pressures are converging and providing an unparalleled framework for progress in health care reform. On the one hand, more than ever before, citizens are demanding that health care providers are prepared to deal effectively with crises like that caused by COVID-19 [3] while providing adequate attention to other health conditions, including post-acute sequelae of COVID-19 (long COVID) [4]. And, on the other hand, health has become a priority in political agendas that must translate one-off experiences into strategic actions at the system level. Despite efforts to maintain health services, disruptions of varying magnitude and duration were found in every type of health services during the pandemic. In addition to implementing innovations in diagnosis, treatment and vaccination technologies [5, 6], to deal with the original virus and the different variants [7], health systems introduced organizational changes to respond to the challenges. They include expanding existing programs capacity, providing outreach to patients at increased risk for adverse outcomes, reorganizing clinical processes and pathways, increasing usage of digital health technologies for regular consultations, reallocating healthcare professionals, optimizing their infrastructure by redesigning existing space for clinical use, or increasing home care [8–12]. Health services must take advantage of the *momentum* generated and move towards a real transformation that will enable them to be high quality entities that maintain or improve the health of their populations. Historically, in healthcare, periods of crisis and adversity have been important catalysts for innovation [13, 14]. But, in addition, the actions and changes that health services had to implement to cope with the pandemic can serve as a basis for learning, improving, and addressing many of the old challenges and those that lay ahead [15]. 

High-quality care implies comprehensive assessment, detection of asymptomatic and co-existing diseases, accurate diagnosis, appropriate and timely treatment, referral when necessary to other care settings, the ability to monitor the patient, and adjust the course of treatment according to the patient’s progress. Equally, equity in access to high quality care must be guaranteed, safeguarding the sustainability of the system [16]. Furthermore, health systems must consider the needs, experiences and preferences of individuals and their right to be treated with respect [17]. In short, we are talking about reforming health systems to achieve better outcomes in what is called the Quadruple Helix (better care for individuals, better health for the population, better experience for clinicians and lower costs).

The Basque Health Care (Osakidetza), is a National Health System (NHS) Beveridge type system with good performance indicators [18], has been highly stressed by the Severe Acute Respiratory Syndrome Corona Virus 2 (SARS-CoV-2) pandemic and has implemented changes to respond to the challenges imposed [19, 20]. The purpose of this work is to identify the main domains behind the relevant changes that could be maintained and/or expanded to contribute to improve quality, efficiency and sustainability of the system in front of the present and future challenges.

## Methods

This qualitative study was carried out in the Basque Country (Spain) (population of 2.2 million), with participants from the Basque Department of Health and the Basque Public Health Service (Osakidetza). The research aimed to understand what, how and why organisational change actions occurred to respond to the demands and challenges that the pandemic brought to health services [21]. Reporting adheres to the Consolidated Criteria for Qualitative Research [22] (Additional file 1. COREQ checklist).

### Study design

A realist design [23] was chosen, focused on the manifest rather than latent interview content, based on qualitative data collection techniques, content analysis and triangulation to identify subject matters organized in themes and sub-themes behind the changes implemented to react to the COVID-19 pandemic in the Basque Country. Key informants were used as an expert source of information.

### Participants

Participants were identified among those with a high level of involvement in the leadership, management, and delivery of public health services (Department of Health and Osakidetza) during the COVID-19 pandemic in the Basque Country (between March and December 2020). Potential informants were selected among professionals with different responsibilities at different levels of the health system who had a professional relationship with the first author (AF). They were selected by convenience sampling, representing different experiential types [24] and interviewed between October and December 2020. Their profiles and expertise covered different perspectives, such as planning, management, information systems, quality, innovation, health service delivery, and social and health care. An introductory email was sent explaining the study by AF, who had a special interest in exploring how health system reform should be oriented as she is the scientific coordinator of the Basque Country’s health services research institute. Interested informants were then invited for an interview by the first author, who presented authors´ interest in the research topic. Recruitment continued until there were enough representatives from all three levels: macro level, Basque Department of Health, and Central Services of the Basque Public Health Service - Osakidetza (*n* = 7); meso, managers of Integrated Health Organisations and Emergencies (*n* = 7); and micro, health professionals (clinicians and nurses) (*n* = 6). Twenty key informants (11 women and 9 men) were contacted and all of them agreed to participate in the study. Prior to the interview, participants were informed about the objectives and characteristics of the study, the confidential handling of data (identity protection) and that participation was voluntary and that they could withdraw from the study at any time without explanation. All interviewees agreed to these terms and gave their consent.

### Interviews

Semi-structured interviews were conducted by teleconference or telephone by AF (PhD, scientific coordinator and senior researcher, female) and IE (BD, junior researcher, female) [25]. The structure of the interviews was based on a prior analysis of the issues in the literature and the authors’ experience [26]. Interview topics, not piloted, explored the redesign of healthcare processes and coordination between healthcare settings, the adaptation of infrastructures and technological services, and the organisation and development of staff during the COVID-19 pandemic (Additional file 2. Interview guide). The ideas expressed by the interviewees were analysed from the perspective of what actions can enhance health system transformation. The participants were alone at their place of work during the interview. Interviews were audio recorded and ranged in length from 30 to 90 min (the average length was 45 min). No interviews were repeated. Field notes were taken during the interviews. Interview transcripts were not returned to participants for comment or correction. No more interviews were hold since data saturation, a notion of informational redundancy, was reached [27]. 

### Analysis

Thematic analysis was done using the *Framework Method.* [28] This approach identifies commonalities and differences in qualitative data, before focusing on the relationships between different parts of the data, thus seeking to draw descriptive conclusions grouped around themes. The analysis was conducted following different stages: familiarisation with the interview, coding, grouping the codes into categories, summarising the data by categories and interpreting the data. To obtain an overall understanding of the content related and to achieve immersion, audio recordings of interviews were listened several times.

Inductive coding of the interview data was performed by AF and IE, and themes and sub-themes were refined throughout the process. During the research, the process of reflexivity was considered, forcing the research team to re-examine their position in relation to the methodology, the theory, the participants, and themselves. Two types of triangulation were used to improve the analysis and interpretation of the findings, and to enhance the credibility of the results. Firstly, data triangulation using various data sources (policy makers, managers, health professionals) was performed. Secondly, triangulation of researchers by involving three researchers to compare, complement and discuss observations, was done [29]. Participants were not asked to provide feedback on the findings.

## Results

**Fig. 1:**
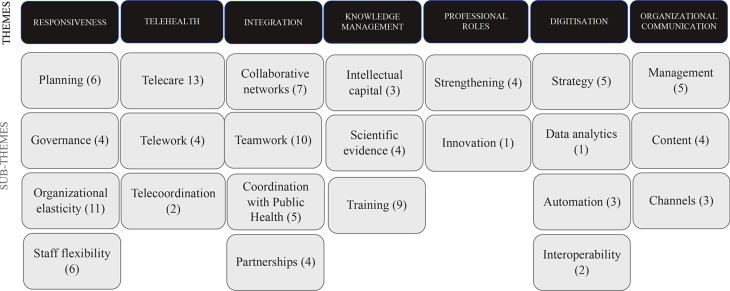
Themes and sub-themes. Seven themes and 24 sub-themes (in brackets) are: responsiveness (planning, governance, organisational elasticity and staff flexibility), telehealth (telecare, telework and telecoordination), integration (teamwork, collaborative networks, coordination with Public Health and partnerships), knowledge management (intellectual capital, scientific evidence, transmission and training), professional roles (strengthening and innovation), digitisation (strategy, data analytics, automation and interoperability) and organisational communication (management, content and channels). Next to each sub-theme, the code number appears in brackets

The analysis of the interviews resulted in the identification of 116 codes, which were reviewed and agreed upon by the researchers. Following the process of methodological analysis, these codes were grouped into different domains: 23 sub-themes, and these in turn into 7 themes (Additional file 3. Codes). Specifically, the themes and sub-themes (in brackets) are: responsiveness (planning, governance, organisational elasticity and staff flexibility), telehealth (telecare, telework and telecoordination), integration (collaborative networks, teamwork, coordination with Public Health and partnerships), knowledge management (intellectual capital, scientific evidence, and training), professional roles (strengthening and innovation), digitisation (strategy, data analytics, automation and interoperability) and organisational communication (management, content and channels) (Fig. 1). Quotations coming from the interviews are framed into the themes and sub-themes which have led to the results described in the following sections (Additional file 4. Quotations).

### Responsiveness

Within the framework of responsiveness, four sub-themes were identified: planning, governance, organisational elasticity, and staff flexibility.

*“Immediacy and uncertainty have meant that the way of working has been reactive. We must not be so reactive; we must hold on to the pull and have tools for decision-making.”* Manager of the Central Services of Basque Public Health Service - Osakidetza.

Planning allows a system to act proactively and cope with different challenges. The ability to plan must be structural and systemic. It facilitates the re-design of organisational models that enable rapid adaptation to changing situations. It comprises testing or simulation of actions in potential scenarios and the development of tools that facilitate decision-making and continuous monitoring of needs. The system must be prepared and organised to deal swiftly, and effectively in uncertain situations where immediacy of response is required. A planning unit within the organisation can assume the aforementioned functions.

*“The fact that there was a single directorate facilitated decision-making and coordination between care areas. The Primary Care teams and middle management acted in an efficient and agile manner.”* Manager of Integrated Healthcare Organization - Osakidetza.

Governance must incorporate an organisation and/or person in a leadership role, with the ability to set direction, define strategies and encourage change and creativity. Participatory governance is essential to ensure coordination between organisations and guarantees working closely and aligned. Enhancing the feeling of an integrated corporation allows for a conversational relationship with different actors responsible for the provision of care. At the same time, the fact that the different areas of care work in unison, facilitates joint decision-making, which translates into agile and effective action by middle management. For example, in Osakidetza, the coordination between the General Directorate and the management teams of the Integrated Healthcare Organisations (IHOs) was facilitated by having a unified Management Executive in IHOs, covering Hospital and Primary Health Care.

*“Mobility of professionals: there must be regulations that allow this, at least a reflection on how resources can be mobilised.”* Clinician.

*“Inter-organisational collaboration is to be valued. Patient flow between organizations, it has been much willingness to collaborate and share knowledge.”* Clinician.

Organisational elasticity means that a health system has the capacity to adapt to dynamic situations. Flexibility can be reflected at different levels. The first refers to the smoothness in restructuring services, i.e., in a natural and frictionless way, for example, health professionals from certain specialties can be available to cover other professional roles if the situation requires it. The second level considers the ability of physical structures to adapt as needed. One case would be the possibility of expanding the number of hospital beds. Another would be the adaptability of spaces, so that they can be used for a different purpose than the original one. The adaptability of infrastructures must be conceived at the time of their construction design. The third level is the flexibility in the provision of care, specifically in terms of location, schedules, and timetables. Thus, the portfolio of services could be structured in such a way that professionals could also carry out their work from home and in time slots other than the pre-established shifts, or by assigning face-to-face and telematic activities to specific schedules. Finally, the fourth is the capacity to promote mobility and the flow of patients between organisations to optimise their care. It allows supply to be dynamically and continuously adapted to demand, not only in terms of volume, but also in terms of type or intensity of care. This avoids peaks of saturation in the organisations where under-utilisation of others coexist. Organisational procedures and regulations should favour such mobility. This would involve, for example, setting up direct corporate circuits in Osakidetza to speed up the referral of seriously ill patients from regional hospitals to tertiary hospitals or a centralized management of Intensive Care Units use.

*“It is necessary to increase the size of the Primary Care nursing staff so that they can take on other tasks in their portfolio of services: control and monitoring of chronic patients, assessment, and monitoring of minor illnesses. This is now forced, but with planning it can be done.”* Manager of Integrated Healthcare Organization - Osakidetza.

Staff flexibility implies having healthcare professionals available when and where they are most needed. It requires proper planning and adaptation of the workforce. It includes designing the portfolio of services for each of the profiles, optimising resources, and assigning functions that add value to the professional and to the patients. Altogether, it will prevent physical-emotional exhaustion and frustration of professionals. It means managing human resources with a flexible and corporate scope. It implies favouring expansion and contraction according to needs, the mobility of professionals, regardless of the place of recruitment, and the adaptation of professional roles and profiles. The expanding role of nurses in care is a clear example. Flexibility requires prior planning and a regulatory framework that allows for adaptations according to demand. Thus, for example, the current procedures for recruiting professionals in the public health system are recognised for their lack of speed. They should be renewed to be able to incorporate professionals in a timely manner.

### Telehealth

In telehealth, three sub-themes were identified: telecare, telework and telecoordination.

*“Telephone appointments are useful. They need to be promoted. Now it is necessary to balance the telephone and face-to-face consultations.”* Clinician.

*“The humanisation of the use of technology must not be lost.”* Nurse.

Telecare comprises remote or non-face-to-face consultations, by telephone or videoconference, and telemedicine. They constitute a novel and potentially beneficial way of providing care for patients, if they meet certain organisational and quality conditions. The management of non-face-to-face consultations (telephone or video-consultation) must be protocolised, systematised and integrated into the usual healthcare activity of the health service. There must be complementarity and flexibility between remote care and face-to-face consultations, establishing criteria at the organisational and corporate levels that establish when each type of care should and should not be used. Moreover, it must be flexible enough to ensure that it is responsive to patients’ preferences and possibilities and does not become a barrier to the provision of accessible, humanised, and equitable care. For example, patients may be unable or unwilling to purchase the devices, may not have the necessary connections, or may not be digitally literate enough. Successful examples of non-face-to-face group sessions of patients, even with very different profiles in terms of technology skills, reinforce the promising future of telecare. Healthcare professionals can assess which type of care (face-to-face or remote) is best for each patient at any given time, depending on their needs and situation. The request for complementary tests, for example, could be managed remotely and, depending on the results of the tests, the professional would assess the format and priority of the next consultation.

*“Teleworking: pros and cons. It is necessary to determine when and when not to telework.”* Manager of the Central Services of Basque Public Health Service - Osakidetza.

At the level of healthcare organisation, telework can allow the relocation of healthcare professionals, while maintaining the same level of quality of care. The introduction of telework in the health system is a valid option for certain profiles and for the completion of certain activities, such as, the review of reports, patient follow-up or telephone consultations. The implementation of telework requires defining which tasks can performed from home, establishing specific timetables, and ensuring the necessary means for professionals.

*“The idea of a call centre formed by nurses and doctors in the care organizations should be retained, homogenised, to provide more accessible and faster care.”* Manager of the Central Services of Basque Public Health Service - Osakidetza.

Telecoordination allows the organisation, in a non-face-to-face manner, of the most appropriate response to citizens’ demands. It is orchestrated around call centres. They can organise the type of response of the health services, offer recommendations for certain health problems that do not require the intervention of a healthcare professional in person, and participate in the follow-up and monitoring of health programmes or help with procedures (appointments, administrative or other). They can be a useful complementary service. For example, a centre staffed by nurses with or without a medical doctor could increase accessibility and speed of care provision. Professional in the call centre could relieve other health actors who are overwhelmed at specific moments, participate in the follow-up of chronic patients, collaborate in the launching of health promotion or prevention programmes, or arrange appointments related to vaccination campaigns such as flu.

#### Integration

In integration, four sub-themes were identified: collaborative networks, teamwork, coordination with Public Health and socio-health, and other partnerships.

*“Organisational barriers between hospital and Primary Care have been broken down. Flexibility is required, avoiding compartmentalisation, working transversally.”* Clinician.

Collaborative networks avoid compartmentalisation and fragmentation of care and to improve system efficiency and continuity of care. It differs from the traditional hierarchical organisational model based on structures. The centralisation of different services, the use of technology and the sophistication of information systems allow a high level of automation and coordination of different activities, favouring agility and accessibility. Thus, for example, the integration of Primary Care and hospitals into Integrated Health Organisations in the Basque Country allows healthcare professionals from different care areas to work together and tackle cross-cutting problems. Another example would be the centralisation of the Osakidetza laboratories, that have functioned as a single operational unit, distributed in different locations. A third one has been the centralised system for purchasing, storing, and distributing material.

*“The professionals have been very flexible; the collaboration model has been exceptional. They have shown great adaptability (e.g. primary care professionals went to hospitals).”* Manager of Integrated Healthcare Organization - Osakidetza.

Teamwork is the collaborative undertaking of tasks by healthcare workers. The ability to work in a team can increase performance and quality of work, increase commitment and boost effort. It is closely linked to professional responsibility and vocational commitment. For example, the experiences of Osakidetza in terms of virtual group activities at management level or clinical sessions between professionals have been clearly positive. The models can be mixed (face-to-face and distance) and can be supported by tools that facilitate interaction. Teamwork fosters communication and nourishes relationships. Thus, for example, thanks to the bonds formed in the work teams, everyday conversations can easily become informal therapeutic sessions. They reach further, where formal corporate emotional support programmes guided by mental healthcare professionals do not reach, providing a space for venting and listening to cope with stressful, and sometimes exhausting, work.

*“Coordination between public health and the care system needs to improve. The shortcomings that existed have become evident. Public health and primary care need to be strengthened.”* Director of the Basque Department of Health.

Coordination with Public Health and social and health care involves using unified, clear, and updated guidelines and criteria for action to promote community-level interventions that respond to complex, mixed, and plural situations. It requires policies, tools, specific procedures, and open and agile communication channels. Thus, for example, coordination between those responsible for Public Health and healthcare providers (especially Primary Care) should be oriented towards gathering information and responding to collective social needs or demands, and creating and maintaining local teams (public health, town councils, educational centres, patient associations, neighbourhood associations, volunteers, etc.). Stable and effective links must also be established between Primary Care and nursing homes, such as, the drawing up of contingency plans in all the Integrated Healthcare Organisations in the Basque Country defining how to provide support to nursing homes, the creation of reference person in nursing homes or the creation of a Directorate for Social and Healthcare Care in the Department of Health.

*“Teams that had never worked together before did so very well. Even with external partners (socio-health centres, prevention services, regional police, municipal police, Institute for Health and Safety at Work…). There was good collaboration, good attitude, willingness. Centrism, origins and interests were forgotten.”* Clinician.

The creation of stable partnerships between administrations with different responsibilities (such as public security, social services, or occupational health) maximises the response capacity and the potential of each of the available assets to face common challenges. An example of this is the joint work carried out by the Basque Government Department of Health and Osakidetza with other departments and institutions (police, town councils, occupational health, etc.).

### Knowledge management

Three sub-themes have been identified in knowledge management: intellectual capital, scientific evidence, and training.

*“The learning and knowledge generated must be integrated into the organisation, standardised and the necessary training articulated.”* Manager of the Central Services of Basque Public Health Service - Osakidetza.

The intellectual capital of the healthcare system is the knowledge (and its value) of professionals including best practices, lessons learned and research-innovation projects. The intellectual growth of an organisation requires optimising its use, knowing, collecting, accessing, and sharing this knowledge. Mechanisms, tools, and procedures must be in place for it to be structured, standardised, and embedded in the organisation. They can be of a varied nature, ranging from questionnaires for collecting information to the well-known *think tanks* that encourage intellectual reflection by providing spaces for dialogue and debate. In Osakidetza, there are experiences of identifying good practices and initiatives promoted by professionals (bottom-up approach).

*“Managing uncertainty: there was a lot of information (info-toxification), a lot of protocols, guidelines etc. We had to try not to lose technical rigour.”* Manager of Integrated Healthcare Organization - Osakidetza.

Scientific evidence is paramount if appropriate decisions are to be made. The ability, when accessing and analysing large volumes of data, to discern relevant, truthful, and up-to-date information, is essential to ensure scientific-technical rigour. Info-toxification and the generation of fake news are phenomena that must be tackled. To this end, it is essential to develop mechanisms that allow information based on scientific evidence to be filtered, structured, and shared in an agile manner. It is critical to establish solid channels of communication with experts in different scientific fields. For example, the implementation of an application for Osakidetza professionals through which a group of experts from different fields provided support in resolving scientific doubts has been valued. Clinical sessions with professionals from different hospitals and different medical specialties, have allowed knowledge-sharing, leading to better patient management and faster decision-making.

Scientific discoveries and clinical practice experience appear in reference journals, scientific society’s media, or professional fora. In a rapidly changing context, organisational mechanisms for transmission are slower than informal circuits. The challenge is to maintain ownership and scientific precision, but to operate with the speed demanded by the situations. One example is the Agile methodologies that help adapting to the conditions of the context, achieving flexibility and immediacy in the response, increasing the quality of the result, the satisfaction of the end user, the motivation of professionals and multidisciplinary collaborative work.

*“Adapt training to the needs of the organisation.”* Clinician.

Training enables experience and knowledge to remain in the organisation. The continuous development of its professional allows the intellectual capital growth. The healthcare system’s training offer must meet the needs of the moment. Predesigned existing continuous education plan has to be adapted to the changing demands and to a more dynamic, personalised format. Versatility increases the ability of professionals to adapt and respond more effectively to different challenges. Transversality is an aptitude in which each professional should be trained, so they have the capacity to extend their functions and reinvent themselves according to needs. Training sessions should be flexible, including both comprehensive programs and short, face-to-face, or virtual (online and offline) training “pills”. Continuous education of professionals cannot be separated from their practice. Professionals must have time in their working day schedule to be trained.

### Professional roles

At the level of professional roles, two sub-themes were identified: strengthening, and innovation.

“*Empowerment of the role of nurses, they can carry out activities assigned to doctors*.” Nurse.

Strengthening certain professional profiles is key to be able to respond to changing and specific demands and become sustainable organisations. It is urgent to give more content and functions to certain groups to maximise and optimise their specific competencies. To this end, it is necessary to analyse what activities each of the professional profiles perform, to assess whether there is another professional category that could perform these tasks more efficiently, and to adapt the service portfolios accordingly. To be able to cover new functions, it is essential to dimension the staff, as well as to train them in the necessary skills and knowledge. For example, nurses can expand their services portfolio and carry out activities traditionally assigned to physicians. They must have a strong and specialised training in clinical and community health. Thus, for example, the accreditation of the speciality of Family and Community Nursing in Spain has been a milestone on the road to strengthening the role of nursing. Another group whose functions could be enriched are ancillary staff, taking care of certain administrative tasks that are currently carried out by doctors.

“*Innovative work teams: customer service area with nursing students to answer questions from the public, 24 h, highly rated*.” Manager of Integrated Healthcare Organization - Osakidetza.

Innovation in the creation, incorporation and utilization of new roles facilitates the entry of new skills into the organisation. It is a matter of integrating new knowledge, expertise, or experience of added value for the organisation. It is an opportunity for growth and allowing the workload of other professionals to be reduced. For example, in the Basque Country there is an under-endowment of professionals such as doctors specialised in preventive medicine, epidemiologists and mathematicians in the healthcare services or healthcare professionals in nursing homes. It would be interesting to innovate to overcome the rigidity of the administration, for example allowing partial dedication of senior healthcare professionals so that they remain active for more years. Experiences such as the User Service Centre made up of nursing students or the incorporation of doctors who are not yet trained as specialists have been very well received.

### Digitisation

This study has identified four sub-themes in digitisation: strategy, data analytics, automation, and interoperability.

*“Strategy for digitisation means an allocated budget, and agility in decision making. The digitisation strategy must come from within the organisation.”* Manager of Integrated Healthcare Organization - Osakidetza.

The digitisation of the healthcare system is a complex process that requires a strategy supported by a solid governance structure with the capacity to define objectives and priorities, establish specific actions and allocate resources and funding. The digitisation strategy must be conceived and orchestrated by corporate management, as well as being contrasted, supported, and executed by digitisation areas or services located in the different organisations of the system. It is essential to analyse and reflect on the digital solutions designed and launched, to evaluate what has happened and what the final service and its integration into the information systems and healthcare practice should be like. For example, contrast groups consisting of experts in specific subjects could perform the validation of these technological solutions (including corporate apps), in addition to studying the sustainability model and dissemination and sales strategies, both in the organisation and in society.

*“There has been coordination with the Ministry of Security. There were no serious issues, control of scams, fishing. When working from home, there is a higher probability of hackers entering. Model to be maintained and more orderly.”* Director of the Basque Department of Health.

Cybersecurity is a substantial pillar in the digitisation process. The establishment of standards, protocols and rules designed to minimise possible risks to the infrastructure and/or the information itself is crucial. Thus, in the Basque Country, consolidating a model of collaboration with the Basque Cybersecurity Centre would be highly recommendable.

*“Data management for prediction and knowledge generation is key.”* Manager of the Central Services of Basque Public Health Service - Osakidetza.

Data analytics capability is critical if the huge volume of data being generated and stored in the healthcare system is to be exploited. Empowerment in the data science field is inevitable if one wants to maximise the performance of the data generated and thus increase knowledge. For example, the evolution and strengthening of Osakidetza’s analytical platform, *Osakidetza Business Intelligence*, would be crucial, to streamline decision-making and ultimately provide higher quality care in a more efficient manner.

*“There are many non-value adding activities where technology is useful. Automate bureaucracy”* Clinician.

Process automation is one of the areas in which technology can be extremely useful. The use of technology allows the healthcare system to move towards the provision of valuable care, understanding value from different perspectives: accessibility, equity and speed for patients and citizens; evidence and quality for professionals; and efficiency and safety for the organisation. The automation of tasks relieves the workload of different professional profiles in the healthcare system, which can devote their efforts to activities of greater value. There are activities that can undoubtedly be automated, such as the bureaucratic and administrative procedures that healthcare professionals perform, triage if certain symptoms appear, and the duration of consultations according to the symptomatology. Thus, for example, the *Interactive Voice Response* (IVR) tool, mostly used in the e-Health centre (health counselling), could be an effective mechanism to speed up certain tasks such as scheduling appointments in screening and vaccination programmes. Similarly, tools based on artificial intelligence, such as the chatbot located on the Osakidetza extranet, which contains a set of questions and answers for citizens, would be very valid.

*“Socio-health record: needs a structural backbone boost, but is not having much effect.”* Director of the Basque Department of Health.

Interoperability of systems is a key field on the way to digitisation. The sharing of information between the systems of the health care provider and the social sector is a pending and urgently needed task. In the Basque Country, timid progress has been made in the development of the socio-health record, and its immediate promotion is a must. Similarly, there are some experiences in which non-public hospitals have accessed Osakidetza’s electronic medical records, allowing access to data on patients whose care has been shared.

### Organisational communication

Within the framework of organisational communication, three sub-themes were identified: management, content, and channels.

*“There has been no corporate communication strategy, and it has been missed. Staff need to be given information that is not on TV. Something corporate.”* Clinician.

The management of communication in the healthcare system includes two distinct audiences as recipients of information: healthcare professionals and the public. From a corporate perspective, fluid and effective communication is required between the health authorities, managers, and healthcare providers, but also with the society, which involves patients,and their families.

*“Truthful, transparent information at the right time is needed.”* Clinician.

The content provided by communication systems must be truthful, transparent, current, and reliable. The aim is to avoid duplicated, misperceived and inopportune information. The existence and implementation of an effective corporate communication strategy is a differentiating element, and it must be coordinated and structured so that its reception by professionals is positive. To provide information that truly reflects reality and is not anecdotal, communication systems must be robust, stable, and reliable, capable of analysing information in real time to guide decision-making.

*“Better channels are needed for smooth communication.”* Manager of the Central Services of Basque Public Health Service - Osakidetza.

The communication channels from the health system to the public must be effective and the messages must reach them directly, correctly defined and without contradictions. The channels used (website, social networks) and the messages must be adapted to their target audience, with an informative strategy aimed at specific groups becoming an alternative to be considered.

The technologies offered by the health system to facilitate the provision of care do not always achieve the expected degree of penetration. The deployment of an effective marketing strategy to make citizens aware of the options offered by the health system is a vital tool. For example, in Osakidetza it is perceived that the campaigns to disseminate the Personal Health Folder, corporate applications, e-Health Centre, etc. should be renewed and re-launched.

## Discussion

Health systems in most countries worldwide have had to cope with the crisis generated by the COVID-19 pandemic for several months. This situation has challenged health services and questioned existing health policies, highlighting several areas where there is room for improvement. Lessons learned during the COVID-19 pandemic can be used not only to respond to potential new pandemic challenges but also to improve quality, efficiency, and sustainability of health systems. Using a realist research design to identify the most relevant concepts behind the adaptation to the COVID-19 pandemic challenges can be an appropriate approach. The *Framework Method* allows in-depth analyses of key themes that can take place across the entire dataset, while the views of each research participant remain connected to other aspects of their narrative. They are persons whose formal roles expose them to the information, knowledge and expertise sought and have absorbed the information in a meaningful way [24, 30]. 

This study has identified dimensions (themes and sub hemes) on which health systems should focus to move forward in their transformation to become high-performing organisations and provide excellent quality care for patients. It is based on the experience of health care managers and professionals in the Basque healthcare service in Spain.

Health systems must have the capacity to act proactively, quickly, and nimbly in the face of different challenges, being able to reconfigure organisational models according to changing needs. Resilient health systems [31] have an up-to-date map of human, physical and information assets, which in turn is the basis for optimal management and care planning. Self-regulation and adaptability are key characteristics of such health systems, being able to effectively mobilise the necessary resources at any given moment [32, 33]. According to several studies, this requires that staffing requirements are anticipated well in advance and systematically considering the needs of populations over time [34, 35], that there is clear legislation that determines the roles and responsibilities of each actor involved [36] and a strong and flexible leadership that fosters communication, coordination and alliances between actors [37, 38]. 

The organisational elasticity of health systems must be reflected in different levels that allow them to adapt to dynamic situations. The possibility of having professionals who can work in different services in an orderly and organised manner is essential. One tool to address capacity shortages and increase operational flexibility is to redeploy staff between units where they are qualified to work, which requires a prior training process. For this, it is imperative that the types of services that can most benefit from such an approach,are identified [39]. Staffing plans that use flexible deployments with a sufficiently high number of staff are able to cope with peaks in demand and are cost-effective, as additional staff can contribute to units that require it [40]. 

The adaptability, convertibility, and scalability of structures in healthcare organisations are also relevant factors favouring organisational elasticity. Flexibility in architectural design must correspond to the complexity and specific transformation timescales of medicine, technology, and spatial organisation of healthcare facilities, as well as to the variability of standards and procedures. The design of space and the organisation of the functioning of the environment can significantly contribute, among other things, reducing the stress experienced by patients, improving the medical outcomes of therapeutic processes, increasing the efficiency of patient care, reducing staff fatigue and stress, and minimising the cost of medical care [41, 42]. Similarly, flexibility in the provision of care, particularly in terms of the working hours of professionals, is a key feature. In fact, studies have shown that the self-scheduling of flexible working hours of healthcare professionals under the supervision of management profiles is beneficial, both from the perspective of professionals’ work-life balance and the quality of care provided to patients [43, 44]. Flexible work organisation, among other issues such as promoting teamwork or reducing administrative burden, has been shown to contribute to greater well-being among professionals, resulting in the provision of higher quality care [45]. 

Enabling the flow of patients between organisations, both from an organisational and regulatory perspective, allows for higher quality, reliability, safety, and efficiency of care when patient load increases significantly. To this end, as several studies have shown, it is necessary to design a system-wide strategy that establishes and enables referral mechanisms that can be activated when necessary [46–48]. 

Telecare can help reorienting the system towards patient-centred care, complementing current patterns of care, reducing overall primary care visits and moderating the associated cost in the long term [49–51]. Benefits also include reduced waiting lists and improved access to care (especially for patients with reduced mobility, those living in nursing homes or in remote geographic areas) [52]. Including telecare into the current care model can contribute to a better use of resources, designing optimal workflows and allowing earlier interventions in the hope of avoiding later clinical events [53]. Establishing which modality of care is appropriate at which time, for which patient group or which has clinical and economic implications, is of key importance [54]. 

Telecare must ensure that the provision of humanised care is not compromised, understood from the perspective of giving equal importance to the social, emotional and psychological needs, as well as the physical needs of patients [55]. Furthermore, as several authors underline, it is imperative that cultural aspects such as privacy and data protection are considered, as these may be the reasons why some patients reject telecare [56]. 

During the pandemic, telework has allowed the relocation of health professionals, being a valid option for certain profiles. Some authors point out that telework has been welcomed by professionals and can be used for activities such as triage, clinical counselling, management and coordination or patient education [57]. Health services can embrace telework provided that cultural issues such as inertia and the innate conservatism of face-to-face working, technological limitations or legal restrictions, are addressed [58].

Telecoordination, which pivots on call centres, is key to providing a rapid and effective response to citizens’ demands. There are experiences of centres where nursing professionals reduce workload of clinical professionals, improve access to primary care, provide greater agility solving health problems, reduce patient travel, and optimize use of emergency services [59]. User perception and satisfaction with call centre performance is positive, mainly due to timely access to quality healthcare [60]. It is crucial that call centre professionals are qualified to undertake the corresponding tasks [61]. 

The relevance of establishing collaborative networks between health care providers with the aim of providing integrated care is widely recognised. Integration of care has a positive impact at different levels [62]. Beyond horizontal integration [63] or vertical integration [64], true integrated organisations have a unity of control and direction that allows them to concentrate all the efforts of their sub-units on the same objectives and strategies. There is a single mission statement, unified ownership, a single hierarchy of authority and a single end result [63, 65]. 

Teamwork has been linked to face-to-face work. However, there are new experiences that point out to tools such as videoconferencing [66, 67] or virtual worlds [68] as effective and useful channels to foster interaction and collaboration between health agents.

The crisis generated by the pandemic provides an opportunity to regenerate the teams formed by healthcare professionals, and thus reduce the number of team failures [69] and the rate of burnout [70, 71]. It is imperative that leaders and managers focus on the team, not just individuals, define and mark the moment of transition, imbuing it with meaning and purpose, and stimulate reflection to enable action [72]. 

This study has shown the need for strong coordination between health services and public health agencies. Achieving substantial and lasting improvements in population health requires a concentrated effort by different entities, aligned with a common goal. The integration of Primary Care and Public Health could enhance the ability of both sectors to perform their respective missions and link with other stakeholders to catalyse a collaborative, cross-sectoral movement towards improving population health [73]. They should be seen as “two interacting and mutually supportive components” of a health system designed to improve the health of populations [74–77]. Similarly, coordination between health and social sector professionals is essential. It requires clarifying roles, negotiating responsibilities and establishing shared accountability, through the development of joint care plans and reflection on the actions expected [78]. 

As described above, proper knowledge management (process of identifying, organising, storing, and disseminating information within an organisation) in healthcare organisations is essential if they are to create, enrich, share, and sustain their intellectual capital. Knowledge mobilisation seeks to strengthen the connections between research, policy and practice, seeking to harness the benefits of research for organisational and societal improvement [79]. It requires encouraging the development of existing capacity; promote the philosophy of “learning by doing”; develop generic capabilities to adapt to change, absorb new knowledge; and facilitate mechanisms that enable the transition between individual, group and organisational learning [80]. 

Having scientific evidence to drive innovation and improvements in care practice is critical [81]. There are significant differences in the systematic use of evidence between current healthcare organisations [82]. Five approaches facilitate a healthcare system to become a learning system: (i) generation of evidence through research; (ii) search for evidence in the literature through dedicated specific staff; (iii) adoption of evidence generated by other organisations; (iv) incorporating clinical decision support systems into practice; and (v) evidence management to procure supplies or equipment provide greater value than others [83]. The pace at which healthcare organisations implement these mechanisms will depend largely on whether there is a perceived return, the availability of resources, and external pressure to be accountable for cost management and quality of care [84]. 

There is no high-quality healthcare system without highly qualified, committed, and motivated professionals. Training of professionals is a priority. The challenge to achieve high competence is having up-to-date education with post-training supervision. Professional management has to support and enforce excellent practice [85]. In addition, clinical education has to instil critical thinking, problem-solving and collaborative relationship skills, promoting adaptability to the context [86]. 

Regarding professional roles, there is a need to reinforce some professional profiles that can take on new responsibilities. Each actor contributes to the efficiency of health systems and to the higher quality of care provided. Primary Care nurses need a greater role so that they can add to their portfolio, activities related to the management and coordination of care for chronic patients. They can use protocols (e.g. adjusting medication for blood pressure or diabetes) and health promotion, as well as providing education and preparation for patients to improve their capacity for self-management and adherence to treatment [87–90]. To this end, it is important to remove existing regulatory barriers, review nursing degree programmes, adjust funding policies according to skills and create sufficient job positions [91]. New profiles with knowledge and experience are of added value for healthcare organisations. Professionals with expertise in mathematics or engineering can contribute significantly to various areas related to healthcare [92]. 

Reference has been made to the relevance of using digital technologies and advanced computer science to strengthen health systems. The design and deployment of a corporate digitalisation strategy is essential [93, 94]. The following pillars stand out as fundamental: strong political commitment and government leadership; a clear regulatory framework that supports secure and effective data exchange; infrastructure provision to enable the deployment of digital solutions; financial investment to drive digitisation; training in the use of innovative digital solutions; research that provides evidence on the impact and cost-effectiveness of digital health interventions; and monitoring and evaluation of the solutions deployed.

Health services are exposed to various cybersecurity threats. It is crucial that healthcare systems prevent cyber-attacks, having well-defined software update procedures, using a virtual local area network, using de-authentication, having a data breach plan, using cloud-based computing, and training employees to be more cybersecurity aware [95, 96]. 

Healthcare systems generate enormous volume of data. Artificial intelligence facilitates, improves, and extends the capacity of data analytics. It can extract the greatest value from the data to support decision making, improve the quality, safety, efficiency of care and patient experience and health outcomes. Some uses are areas where machines can outperformed humans (cancer screening or diabetic retinopathy monitoring), tasks where errors do not have serious consequences (flagging an at-risk population for vaccination), or situations where medical professionals are not available but a machine can do a good job (using a chatbot to show a patient how to give an insulin injection) [97, 98]. Machine learning, natural language processing, voice technologies and intelligent assistants can transform electronic medical records from systems of records to systems of intelligence [99, 100]. If the potential of health data is to be maximised, data sources must be mandatorily interoperable, a prerequisite for most innovations envisaged in future medicine [101]. 

Effective organisational communication requires a reciprocal process of individuals sending and receiving accurate information that shapes and reshapes people’s attitudes, behaviours, and cognitions [102]. This work has underlined the relevance of the content being communicated being truthful, transparent, current, and authoritative, and reaching the intended target populations. Oher authors point out the relevance of establishing mechanisms to overcome cognitive barriers (where the exchange of information is interrupted, contains insufficient information, or lacks context) or linguistic barriers (where the structure and form of the components) [103], as well as establishing procedures to avoid misinformation mainly disseminated on social networks [104, 105]. 

## Conclusions

To counter current and future challenges, the transformation of health systems into organisations that can improve the health of their populations and providing high quality care is crucial. Such a transformation requires multidimensional and comprehensive reform, encompassing both strategic and operational actions in diverse areas and a broad and sustained political, technical, and financial commitment. This study provides evidence on the levers on which health systems and policy makers need to focus, and which policy makers need to ground in specific actions tailored to the particularities of their contexts.

### Electronic supplementary material

Below is the link to the electronic supplementary material.

Supplementary Material 1

Supplementary Material 2

Supplementary Material 3

Supplementary Material 4

## Data Availability

The datasets used and/or analysed during the current study are available from the corresponding author on reasonable request.
