# Supplementary material for: Transforming health care systems towards high-performance organizations: qualitative study based on learning from COVID-19 pandemic in the Basque Country (Spain)
Source: BMC Health Serv Res. 2024 Mar 21;24:364. doi: 10.1186/s12913-024-10810-w (PMC10958960; doi:10.1186/s12913-024-10810-w)
Supplement: Supplementary file 1 — Supplementary Material 1 [file 12913_2024_10810_MOESM1_ESM.docx]

**Article title:** Transforming health care systems towards high-performance organizations: learning from COVID-19 pandemic in the Basque Country

**Journal name:** BMC Health Services Research

**Authors information:**

1. **Ane Fullaondo** * (Corresponding author)

Affiliation: Kronikgune Institute for Health Services Research, Barakaldo, Bizkaia, Spain.

Email: afullaondo@kronikgune.org

1. **Irati Erreguerena**

Affiliation: Kronikgune Institute for Health Services Research, Barakaldo, Bizkaia, Spain.

Email: ierreguerena@kronikgune.org

1. **Esteban de Manuel**

Affiliation: Kronikgune Institute for Health Services Research, Barakaldo, Bizkaia, Spain.

Email: edemanuel@kronikgune.org

**Interview guide**

|  | **Part I: Identification** | | |
| --- | --- | --- | --- |
| Identification Code: __________ | | | Date: _____ |
| Data collector name: ______________ | | |  |
|  | **Part II: Socio-demographic characteristics** | | |
| **S.no** | **Question/s** | **Response** | |
|  | Gender | 1. Male 2. Female | |
|  | Job position | 1. Macro level (Basque Department of Health, and Central Services of the Basque Public Health Service)  2. Meso level ( managers of Integrated Health Organizations and Emergencies)  3. Micro level (health professionals, clinicians and nurses) | |
|  | **Part III:**  **Changes and organization of procedures in different areas** | | |
|  | What type of changes in the EMERGENCY DEPARTMENT have been made in response to the COVID-19 pandemic? |  | |
|  | Regarding EMERGENCY DEPARTMENT, which of these changes do you think should be maintained or promoted in order to transform the health system? |  | |
|  | What type of changes in the HOME CARE and PRIMARY CARE have been made in response to the COVID-19 pandemic? |  | |
|  | Regarding HOME CARE and PRIMARY CARE, which of these changes do you think should be maintained or promoted in order to transform the health system? |  | |
|  | What type of changes have been made with regards to new COORDINATION PROCESSES between primary care, hospital and emergency department in response to the COVID-19 pandemic? |  | |
|  | Regarding COORDINATION between care settings, which of these changes do you think should be maintained or promoted in order to transform the health system? |  | |
|  | **Part IV: Changes and organization of infrastructure and resource allocation** | | |
|  | What type of changes with regards to INFORMATION AND COMMUNICATION TECHNOLOGIES (medical record functionalities, health folder, Artificial Intelligence, Internet of Things, etc.) have been made in response to the COVID-19 pandemic? |  | |
|  | Regarding INFORMATION AND COMMUNICATION TECHNOLOGIES (medical record functionalities, health folder, Artificial Intelligence, Internet of Things, etc.), which of these changes do you think should be maintained or promoted in order to transform the health system? |  | |
|  | What type of changes with regards to REMOTE MONITORING AND VIRTUAL VISITS (technologies -telephone, messaging, online platforms, telemedicine, others and the organizational circuits) in place have been made in response to the COVID-19 pandemic? |  | |
|  | Regarding REMOTE MONITORING AND VIRTUAL VISITS (technologies -telephone, messaging, online platforms, telemedicine, others and the organizational circuits), which of these changes do you think should be maintained or promoted in order to transform the health system? |  | |
|  | **Part V: Staff change, organization and development (including management of staff shortages)** | | |
|  | What type of changes with regards to RECRUITMENT, HIRING, and REPLACEMENT IN CASE OF ILLNESS have been made in response to the COVID-19 pandemic? |  | |
|  | Regarding RECRUITMENT, HIRING, and REPLACEMENT, which of these changes do you think should be maintained or promoted in order to transform the health system? |  | |
|  | What type of changes with regards to WORKING HOURS and SHIFT CHANGES have been made in response to the COVID-19 pandemic? |  | |
|  | Regarding WORKING HOURS and SHIFT CHANGES, which of these changes do you think should be maintained or promoted in order to transform the health system? |  | |
|  | What type of changes with regards to OVERLOAD MANAGEMENT MODELS have been made in response to the COVID-19 pandemic? |  | |
|  | Regarding OVERLOAD MANAGEMENT MODELS, which of these changes do you think should be maintained or promoted in order to transform the health system? |  | |
|  | What type of changes with regards to EXTENSION OR CHANGE OF PROFESSIONAL ROLES have been made in response to the COVID-19 pandemic? |  | |
|  | Regarding EXTENSION OR CHANGE OF PROFESSIONAL ROLES, which of these changes do you think should be maintained or promoted in order to transform the health system? |  | |
|  | What type of changes with regards to TRAINING IN NEW PROCEDURES have been made in response to the COVID-19 pandemic? |  | |
|  | Regarding TRAINING IN NEW PROCEDURES, which of these changes do you think should be maintained or promoted in order to transform the health system? |  | |
|  | What type of changes with regards to SUPPORT TO PROFESSIONALS have been made in response to the COVID-19 pandemic? |  | |
|  | Regarding SUPPORT TO PROFESSIONALS, which of these changes do you think should be maintained or promoted in order to transform the health system? |  | |
|  | What type of changes with regards to SUPPORT TO INFORMAL CAREGIVERS have been made in response to the COVID-19 pandemic? |  | |
|  | Regarding SUPPORT TO INFORMAL CAREGIVERS, which of these changes do you think should be maintained or promoted in order to transform the health system? |  | |
