# Supplementary material for: Transforming health care systems towards high-performance organizations: qualitative study based on learning from COVID-19 pandemic in the Basque Country (Spain)
Source: BMC Health Serv Res. 2024 Mar 21;24:364. doi: 10.1186/s12913-024-10810-w (PMC10958960; doi:10.1186/s12913-024-10810-w)
Supplement: Supplementary file 2 — Supplementary Material 2 [file 12913_2024_10810_MOESM2_ESM.docx]

**Article title:** Transforming health care systems towards high-performance organizations: learning from COVID-19 pandemic in the Basque Country

**Journal name:** BMC Health Services Research

**Authors information:**

1. **Ane Fullaondo** * (Corresponding author)

Affiliation: Kronikgune Institute for Health Services Research, Barakaldo, Bizkaia, Spain.

Email: afullaondo@kronikgune.org

1. **Irati Erreguerena**

Affiliation: Kronikgune Institute for Health Services Research, Barakaldo, Bizkaia, Spain.

Email: ierreguerena@kronikgune.org

1. **Esteban de Manuel**

Affiliation: Kronikgune Institute for Health Services Research, Barakaldo, Bizkaia, Spain.

Email: [edemanuel@kronikgune.org](mailto:edemanuel@kronikgune.org)

| Theme | Subtheme | Codes | No. |
| --- | --- | --- | --- |
| Responsiveness | Planning | Proactivity, responsiveness, organizational planning, establishment of a planning unit, generational handover planning, flexibility and adaptability of the systems | 6 |
|  | Governance | Sense of corporateness, utility of structural integration, participation in decision-making, leadership | 4 |
|  | Organisational elasticity | Regulatory agility, patient flow between organizations, flexibility in HR management, collaboration between organizations, regulation to manage the movement of professionals, flexibility and adaptability of the system, adaptability of physical structures, physical spaces, timetables, physical spaces, frustration and stress | 11 |
|  | Staff flexibility | Regulatory mechanisms to streamline procurement, regulatory mechanisms to expedite recruitment, staffing sizing, staffing levels, depletion, sizing of infrastructure | 6 |
| Telehealth | Telecare | Implementation of the mixed model, criteria for use and incorporation into service portfolios, management of activities, acceptance among professionals, patient perception, humanization, acceptance by citizens, telemedicine, inequality, flexibility of supply, approach, access, digital literacy | 13 |
|  | Telework | Criteria for defining the telework model, activities to be carried out in teleworking, equipment needed for teleworking, acceptance of the technology | 4 |
|  | Telecoordination | Utility of administrative call centre, creation of health call centres | 2 |
| Integration | Collaborative networks | Usefulness of structural integration, sense of belonging, system as a network, homogenization in laboratory procedures, sophistication of the laboratory, centralization of services, relocation of problems | 7 |
|  | Teamwork | Usefulness and advantages, criteria for use, collaboration between professionals, adaptability of professionals, vocation, responsibility of professionals, commitment, formal support programmes, informal support, community support | 10 |
|  | Coordination with Public Health | Linking Public Health to the health system, coordination between Public Health and Primary Care, unification of criteria, streamlined communication, multidisciplinary teams | 5 |
|  | Partnerships | Building alliances, collaboration between the social and health care sectors, coordination between nursing homes and primary care, governance in the social and healthcare field | 4 |
| Knowledge management | Intellectual capital | Knowledge integration and structuring, systematic identification of good practices, research, innovation, exploitation of knowledge | 3 |
|  | Scientific evidence | Ensuring scientific and technical rigor, support from professional experts, agility in the generation and sharing of knowledge, usefulness and advantages | 4 |
|  | Training | Adequacy of training, training format, streamlined registration of training, facilitating spaces for training, capacity of professionals to respond to different challenges (adaptation), planning of roles and their scope of action, polyvalence of roles, ability to adapt, flexibility of roles | 9 |
| Professional roles | Strengthening | Empowerment, empowering nursing, empowering the role of the administrative area, recognition and exigency | 4 |
|  | Innovation | Incorporation of new roles | 1 |
| Digitisation | Strategy | Funding, digitisation unit, evaluation, security, promotion of corporate apps | 5 |
|  | Data analytics | Data exploitation | 1 |
|  | Automation | Need, automatable activities, tools | 3 |
|  | Interoperability | Shared health records, socio-health record | 2 |
| Organisational communication | Management | Difficulty in communicating between structures and actors, dissemination and marketing of existing technological solutions, corporate communication strategy, ability in communication skills, relevance | 5 |
|  | Content | Messages, communication channels, clarity, transparency and truthfulness | 4 |
|  | Channels | Capturing information to facilitate decision-making, communication channels, agility of communication | 3 |
