# Supplementary material for: Transforming health care systems towards high-performance organizations: qualitative study based on learning from COVID-19 pandemic in the Basque Country (Spain)
Source: BMC Health Serv Res. 2024 Mar 21;24:364. doi: 10.1186/s12913-024-10810-w (PMC10958960; doi:10.1186/s12913-024-10810-w)
Supplement: Supplementary file 3 — Supplementary Material 3 [file 12913_2024_10810_MOESM3_ESM.docx]

**Article title:** Transforming health care systems towards high-performance organizations: learning from COVID-19 pandemic in the Basque Country

**Journal name:** BMC Health Services Research

**Authors information:**

1. **Ane Fullaondo** * (Corresponding author)

Affiliation: Kronikgune Institute for Health Services Research, Barakaldo, Bizkaia, Spain.

Email: afullaondo@kronikgune.org

1. **Irati Erreguerena**

Affiliation: Kronikgune Institute for Health Services Research, Barakaldo, Bizkaia, Spain.

Email: ierreguerena@kronikgune.org

1. **Esteban de Manuel**

Affiliation: Kronikgune Institute for Health Services Research, Barakaldo, Bizkaia, Spain.

Email: edemanuel@kronikgune.org

**Consolidated criteria for reporting qualitative studies (COREQ): 32-item checklist**

Developed from:

Tong A, Sainsbury P, Craig J. Consolidated criteria for reporting qualitative research (COREQ): a 32-item checklist for interviews and focus groups. *International Journal for Quality in Health Care*. 2007. Volume 19, Number 6: pp. 349 – 357

| **No. Item** | **Guide questions/description** | **Reported on Page #** |
| --- | --- | --- |
| DOMAIN 1: RESEARCH TEAM AND REFLEXIVITY |  |  |
| Personal Characteristics |  |  |
| 1. Inter viewer/facilitator | Which author/s conducted the interview or focus group? | Page 6, lines 142-144 |
| 2. Credentials | What were the researcher’s credentials? E.g. PhD, MD | Page 6, lines 142-144 |
| 3. Occupation | What was their occupation at the time of the study? | Page 6, lines 142-144 |
| 4. Gender | Was the researcher male or female? | Page 6, lines 142-144 |
| 5. Experience and training | What experience or training did the researcher have? | Page 6, lines 142-144 |
| Relationship with participants |  |  |
| 6. Relationship established | Was a relationship established prior to study commencement? | Page 5, line 122 |
| 7. Participant knowledge of the interviewer | What did the participants know about the researcher? e.g. personal goals, reasons for doing the research | Page 5, lines 127-129 |
| 8. Interviewer characteristics | What characteristics were reported about the inter viewer/facilitator? e.g. Bias, assumptions, reasons and interests in the research topic | Page 5, lines 127-129 |

| DOMAIN 2: STUDY DESIGN |  |  |
| --- | --- | --- |
| Theoretical framework |  |  |
| 9. Methodological orientation and Theory | What methodological orientation was stated to underpin the study? e.g. grounded theory, discourse analysis, ethnography, phenomenology, content analysis | Page 5, lines 111-112  Page 6, lines 158-163 |
| Participant selection |  |  |
| 10. Sampling | How were participants selected? e.g. purposive, convenience, consecutive, snowball | Page 5, line 123 |
| 11. Method of approach | How were participants approached? e.g. face-to-face, telephone, mail, email | Page 5, line 126 |
| 12. Sample size | How many participants were in the study? | Page 5, line 135 |
| 13. Non-participation | How many people refused to participate or dropped out? Reasons? | Page 5, line 139-140 |
| Setting |  |  |
| 14. Setting of data collection | Where was the data collected? E.g. home, clinic, workplace | Page 6, line 151 |
| 15. Presence of non-participants | Was anyone else present besides the participants and researchers? | Page 6, line 151 |
| 16. Description of sample | What are the important characteristics of the sample? e.g. demographic data, date | Page 5, line 131-135 |
| Data collection |  |  |
| 17. Interview guide | Were questions, prompts, guides provided by the authors? Was it pilot tested? | Additional file 2. Interview guide |
| 18. Repeat interviews | Were repeat inter views carried out? If yes, how many? | Page 6, line 153 |
| 19. Audio/visual recording | Did the research use audio or visual recording to collect the data? | Page 6, line 152 |
| 20. Field notes | Were ﬁeld notes made during and/or after the inter view or focus group? | Page 6, lines 153 |
| 21. Duration | What was the duration of the inter views or focus group? | Page 6, lines 152-153 |
| 22. Data saturation | Was data saturation discussed? | Page 6, 155-156 |
| 23. Transcripts returned | Were transcripts returned to participants for comment and/or correction? | Page 6, line 154 |
| DOMAIN 3: ANALYSIS AND FINDINGS |  |  |
| Data analysis |  |  |
| 24. Number of data coders | How many data coders coded the data? | Page 6, line 167-168 |
| 25. Description of the coding tree | Did authors provide a description of the coding tree? | Additional file 3. Codes |
| 26. Derivation of themes | Were themes identiﬁed in advance or derived from the data? | Page 6, lines 167-168 |
| 27. Software | What software, if applicable, was used to manage the data? | NA |
| 28. Participant checking | Did participants provide feedback on the ﬁndings? | Page 7, line 176 |
| Reporting |  |  |
| 29. Quotations presented | Were participant quotations presented to illustrate the themes/ﬁndings? Was each quotation identiﬁed? e.g. participant number | Additional file 4. Quotations and along the manuscript |
| 30. Data and ﬁndings consistent | Was there consistency between the data presented and the ﬁndings? | Results section |
| 31. Clarity of major themes | Were major themes clearly presented in the ﬁndings? | Results section |
| 32. Clarity of minor themes | Is there a description of diverse cases or discussion of minor themes? | Diverse cases were not found during the interviews |
