# Supplementary material for: Transforming health care systems towards high-performance organizations: qualitative study based on learning from COVID-19 pandemic in the Basque Country (Spain)
Source: BMC Health Serv Res. 2024 Mar 21;24:364. doi: 10.1186/s12913-024-10810-w (PMC10958960; doi:10.1186/s12913-024-10810-w)
Supplement: Supplementary file 4 — Supplementary Material 4 [file 12913_2024_10810_MOESM4_ESM.docx]

| **THEMES** | **SUB-THEMES** | **QUOTATIONS** | **INTERVIEWEES´ PROFILE** |
| --- | --- | --- | --- |
| Responsiveness | Planning  Governance  Organisational elasticity  Staff flexibility | *“Immediacy and uncertainty have meant that the way of working has been reactive. We must not be so reactive; we must hold on to the pull and have tools for decision-making.”*  *“In a structure where there are planners and care provisioners, there must be prior "training/rehearsal" in order to be able to react in an agile and rapid manner. It has to be thought out how to set up the necessary mechanisms.”*  *“At the information systems level, the covid unit, which carries out monitoring, has been created. This should be the embryo of the planning unit.”*  *“The fact that there was a single directorate facilitated decision-making and coordination between care areas. The Primary Care teams and middle management acted in an efficient and agile manner.”*  *“Leadership/non-leadership… There has to be a "captain" to set direction, strategy. Whether it is a person or an entity/organisation.”*  *“The most important thing has been participation and collaboration in governance (board of directors, working groups, commissions). Culture to involving everyone at every step and gathering proposals already existed but it became a daily practice.”*  *“The system is very rigid, and when there is alarm it is difficult to move. We need regulatory procedures that allow for movement between areas.”*  *“Mobility of professionals: there must be regulations that allow this, at least a reflection on how resources can be mobilised.”*  *“Inter-organisational collaboration is to be valued. Patient flow between organizations, it has been much willingness to collaborate and share knowledge.”*  *“Structures should expand, and contract as needed and should be thought through in construction.”*  *“Flexibilisation of timetables, the offer has to be structured differently.”*  *“Extending working hours and ensuring that the place of work is not only the consulting room. Other spaces can be used for other purposes. Versatility of professionals to do their work in other places.”*  *“It is necessary to increase the size of the Primary Care nursing staff so that they can take on other tasks in their portfolio of services: control and monitoring of chronic patients, assessment and monitoring of minor illnesses. This is now forced, but with planning it can be done.”*  *“During the state of alarm it has been possible to recruit by qualification level and assign tasks accordingly (graduates in medicine). This should be maintained but the change of regulations is very slow and rigid.”* | *Manager of the Central Services of Basque Public Health Service – Osakidetza*  *Manager of the Central Services of Basque Public Health Service – Osakidetza*  *Manager of the Central Services of Basque Public Health Service – Osakidetza*  *Manager of Integrated Healthcare Organization – Osakidetza*  *Manager of the Central Services of Basque Public Health Service – Osakidetza*  *Manager of Integrated Healthcare Organization – Osakidetza*  *Manager of Integrated Healthcare Organization – Osakidetza*  *Clinician*  *Clinician*  *Manager of the Central Services of Basque Public Health Service – Osakidetza*  *Manager of Integrated Healthcare Organization – Osakidetza*  *Manager of Integrated Healthcare Organization – Osakidetza*  *Manager of Integrated Healthcare Organization – Osakidetza*  *Manager of Integrated Healthcare Organization – Osakidetza* |
| Telehealth | Telecare  Telework  Telecoordination | *“Telephone appointments are useful. They need to be promoted. Now it is necessary to balance the telephone and face-to-face consultations.”*  *“The humanisation of the use of technology must not be lost.”*  *“Clustering patients, adapting to patients’ needs, those who want digital and those who don’t, traditional channels.”*  *“Telemonitoring services are useful, installing equipment at home to measure vital signs and send the information to healthcare professionals.”*  *“Teleworking: pros and cons. It is necessary to determine when and when not to telework.”*  *“Teleworking for telephone consultations, review of medical records, patient follow-up…”*  *“The administrative user service centre is used to give appointments for flu vaccinations. Now Osakidetza calls people to negotiate the appointment (proactivity). This proactivity can help to make follow-ups in other cases, e.g. chronic patients.”*  *“The idea of a call centre formed by nurses and doctors in the care organizations should be retained, homogenised, to provide more accessible and faster care.”* | *Clinician*  *Nurse*  *Nurse*  *Clinician*  *Manager of the Central Services of Basque Public Health Service – Osakidetza*  *Manager of Integrated Healthcare Organization – Osakidetza*  *Manager of the Central Services of Basque Public Health Service – Osakidetza*  *Manager of the Central Services of Basque Public Health Service – Osakidetza* |
| Integration | Collaborative networks  Teamwork  Coordination with Public Health and social and health care settings  Partnerships | *“The integration between primary care and hospital has been useful to be more agile in the design of new circuits.”*  *“Organisational barriers between hospital and Primary Care have been broken down. Flexibility is required, avoiding compartmentalisation, working transversally.”*  *“Strengthening Osakidetza as a network. There has been a boost.”*  *“The centralised material distribution system, the centralisation of purchases and having a large warehouse is very positive and agile.”*  *“Professionals have a vocation, if they see a need they get involved… necessity, urgency, motivation, commitment.”*  *“The professionals have been very flexible; the collaboration model has been exceptional. They have shown great adaptability (e.g. primary care professionals went to hospitals).”*  *“Group activity: virtual meetings. We will have to see what it is for and what it is not for. Communication through technology is here to stay. It is more efficient to hold virtual meetings (fewer hours, less risk). Mixed models between professionals (virtual and face-to-face).”*  *“Coordination between public health and the care system needs to improve. The shortcomings that existed have become evident. Public health and primary care need to be strengthened.”*  *“Widespread feeling of having to coordinate better with nursing homes. The new health and social care directorate can be helpful in moving forward.”*  *“A contingency plan has been drawn up in all the healthcare organizations on how to support the nursing homes. Proximity referents have been created in the nursing homes.”*  *“Teams that had never worked together before did so very well. Even with external partners (socio-health centres, prevention services, regional police, municipal police, Institute for Health and Safety at Work...). There was good collaboration, good attitude, willingness. Centrisms, origins and interests were forgotten.”* | *Manager of Integrated Healthcare Organization – Osakidetza*  *Clinician*  *Director of the Basque Department of Health*  *Director of the Basque Department of Health*  *Manager of Integrated Healthcare Organization – Osakidetza*  *Manager of Integrated Healthcare Organization – Osakidetza*  *Manager of the Central Services of Basque Public Health Service – Osakidetza*  *Director of the Basque Department of Health*  *Director of the Basque Department of Health*  *Director of the Basque Department of Health*  *Clinician* |
| Knowledge management | Intellectual capital  Scientific evidence  Training | *“The learning and knowledge generated must be integrated into the organisation, standardised and the necessary training articulated.”*  *“Accessing research and innovation projects (bottom up) is important.”*  *“Managing uncertainty: there was a lot of information (info-toxification), a lot of protocols, guidelines etc. We had to try not to lose technical rigour.”*  *“It was solved in the best possible way. Contact was made with other scientific groups who had other sources.”*  *“Online clinical sessions involving other hospitals. It has been important to have well-structured information.”*  *“Adapt training to the needs of the organisation.”*  *“It is important to create new profiles or extend existing ones, although the system is very constrained.”*  *“It is necessary to reserve time in daily agendas to be able to carry out training activities.”* | *Manager of the Central Services of Basque Public Health Service – Osakidetza*  *Manager of the Central Services of Basque Public Health Service – Osakidetza*  *Manager of Integrated Healthcare Organization – Osakidetza*  *Manager of Integrated Healthcare Organization – Osakidetza*  *Manager of Integrated Healthcare Organization – Osakidetza*  *Clinician*  *Nurse*  *Clinician* |
| Professional roles | Strengthening  Innovation | *“Empowerment of the role of nurses, they can carry out activities assigned to doctors.”*  *“It is a priority to give the customer service area more roles. They can do administrative tasks that are currently done by doctors: reports, queries about appointments, distinguishing which appointments are for nurses and which are for doctors, etc. They need more support, staff sizing.”*  *“Innovative work teams: customer service area with nursing students to answer questions from the public, 24h, highly rated.”*  *“Because of the rigidity of the administration, articulating non-full time working hours, senior work etc. is very difficult. Intermediate compromises could be made to prevent people from retiring or pre-retirement.”* | *Nurse*  *Manager of Integrated Healthcare Organization – Osakidetza*  *Manager of Integrated Healthcare Organization – Osakidetza*  *Manager of Integrated Healthcare Organization – Osakidetza* |
| Digitisation | Strategy  Cybersecurity  Data analytics  Automation  Interoperability | *“Strategy for digitisation means an allocated budget, and agility in decision making. The digitisation strategy must come from within the organisation.”*  *“Digitalisation is very important. It has accelerated, you have to assess what has happened to know what has worked well.”*  *“Contrast group - validate that the designed solution works. You have to get it right, which model and how it is integrated.”*  *“There has been coordination with the Ministry of Security. There were no serious issues, control of scams, fishing. When working from home, there is a higher probability of hackers entering. Model to be maintained and more orderly.”*  *“Data management for prediction and knowledge generation is key.”*  *“There are many non-value adding activities where technology is useful. Automate bureaucracy”*  *“Automated triage, there are protocols that can be automated.”*  *“Socio-health record: needs a structural backbone boost, but is not having much effect.”* | *Manager of Integrated Healthcare Organization – Osakidetza*  *Manager of the Central Services of Basque Public Health Service – Osakidetza*  *Clinician*  *Director of the Basque Department of Health*  *Manager of the Central Services of Basque Public Health Service – Osakidetza*  *Clinician*  *Nurse*  *Director of the Basque Department of Health* |
| Organisational communication | Management  Content  Channels | *“There has been no corporate communication strategy, and it has been missed. Staff need to be given information that is not on TV. Something corporate.”*  *“Deficits in communication between professionals and with society.”*  *“Communication with families and patients’ needs to be improved.”*  *“There have been duplicate, misperceived and untimely orders.”*  *“Truthful, transparent information at the right time is needed.”*  *“Better channels are needed for smooth communication.”* | *Clinician*  *Clinician*  *Nurse*  *Clinician*  *Manager of the Central Services of Basque Public Health Service – Osakidetza* |
